# Orf Infection on the Scalp of a Taiwanese Woman: A Case Report and Literature Review

**DOI:** 10.3390/life13020358

**Published:** 2023-01-28

**Authors:** Shiow-Jen Juang, Khin-Than Win, Yen-Lin Chen, Hung-Wen Chen, Pai-Shan Cheng

**Affiliations:** 1 Department of Dermatology, Chi Mei Medical Center, Tainan 71004, Taiwan; 2 Department of Pathology, Chi-Mei Medical Center, Tainan 71004, Taiwan; 3 Tri-Service General Hospital, Taipei 11490, Taiwan; 4 Graduate Institute of Applied Science and Engineering, Fu-Jen Catholic University, New Taipei 242, Taiwan

**Keywords:** Orf, Orf virus, ecthyma contagiosum, zoonosis, Parapoxvirus

## Abstract

Background: Orf, or ecthyma contagiosum, is a zoonosis caused by Parapoxvirus that infects sheep and goats. Human transmission typically occurs in persons in contact with the infected animals or contaminated fomites and environment. In humans, it generally occurs as solitary or multiple skin lesions on the hands or fingers. Involvement of the head region has rarely been reported. Case presentation: We report an unusual case with multiple orf lesions on the scalp of a middle-aged woman, along with a review of previously reported Orf cases on the head region. Conclusions: Although Orf infection rarely happens on the head region, it should be considered in the differential diagnosis of cases with relevant animal exposure.

## 1. Introduction

Orf, also known as ecthyma contagiosum, is a worldwide zoonosis caused by Orf virus, which belongs to the genus Parapoxvirus. Orf is endemic in livestock such as sheep and goats and is usually transmitted to humans who are in contact with the infected animals. Less commonly, humans can also be infected after contact with contaminated fomites and environments. It usually causes solitary or several skin lesions over exposed areas, with most of the lesions occurring on the fingers, hands, forearms, or face. [1] Orf lesions on the scalp have rarely been reported in the literature [2,3]. Herein, we report an unusual presentation of multiple Orf nodules on the scalp, as well as a literature review of previously reported Orf cases on the head region.

## 2. Case Report

**Figure 1:**
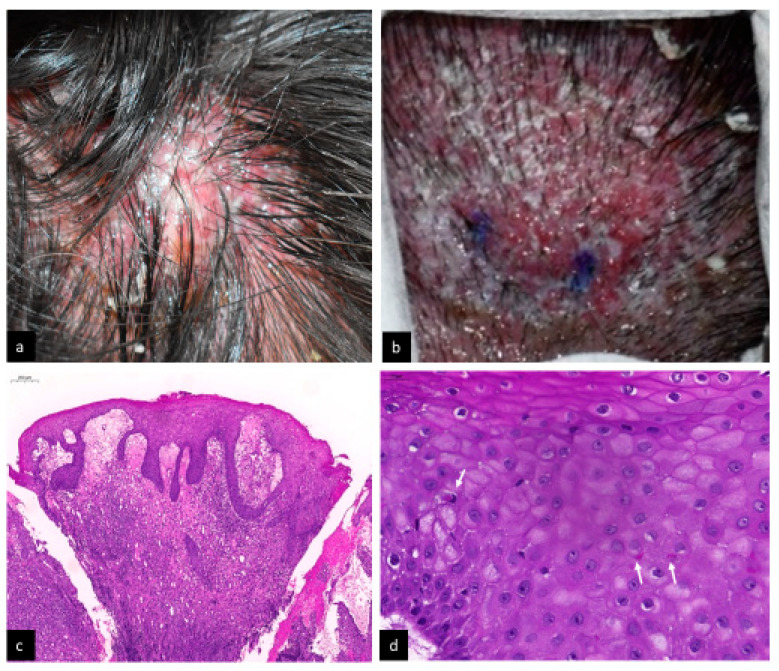
(**a**) One of the four large erythematous, weeping, and infiltrated noduloplaque with surface cobblestone appearance over scalp; (**b**) close-up view of the 4 × 4 cm noduloplaque over vertex scalp; (**c**) the epidermis revealed acanthosis with finger-like downward projections. Prominent papillary dermal edema and heavy lymphohistiocytic infiltration extended from upper dermis to reticular dermis (H&E, ×50); and (**d**) koilocytic change of the upper epidermis and intracytoplasmic eosinophilic inclusions (white arrows) in vacuolated epidermal cells (H&E, ×400).

A 55-year-old Taiwanese woman had a history of Hailey—Hailey disease with some stable erythematous macerated patches over bilateral axillary and submammary areas without regular treatment. She presented with four large (size ranged from 2 × 2 cm to 4 × 4 cm), itchy, tendered, erythematous, weeping, and infiltrated noduloplaques with a surface cobblestone appearance over the vertex and occipital scalp for 5 days (Figure 1a,b). Skin biopsy was performed under the impression of atypical infection and oral minocycline and topical gentamicin ointment were administered. A histologic examination revealed surface erosions with serum crusts, papillomatosis, and acanthosis with finger-like downward projections of the epidermis. The dermal changes included prominent papillary dermal edema and heavy lymphohistiocyte infiltration extending from the upper dermis to the reticular dermis (Figure 1c). Cytoplasmic vacuolation (koilocytic change) of the upper epidermis and intracytoplasmic eosinophilic inclusions in vacuolated epidermal cells (Figure 1d) were also noted. No acantholysis change related to Hailey—Hailey disease was noted. Special stains, including Grocott-Gomori’s methenamines silver (GMS), periodic acid-Schiff (PAS), and acid-fast stain all revealed negative results. The tissue Gram stain and fungal and mycobacterial culture were also negative.

One week later, her skin lesions improved partially with less swelling and oozing, but was still indurated with some yellowish crusts. On questioning, the patient reported that she was a farmworker that raised goats and sold goat milk. Many lambs had some skin lesions located at the muzzle area during that period. Since Orf infection was highly suspected based on the occupational history and consistent histologic findings, we further arranged a polymerase chain reaction (PCR) analysis of the formalin-fixed paraffin-embedded skin biopsy specimens.

**Figure 2:**
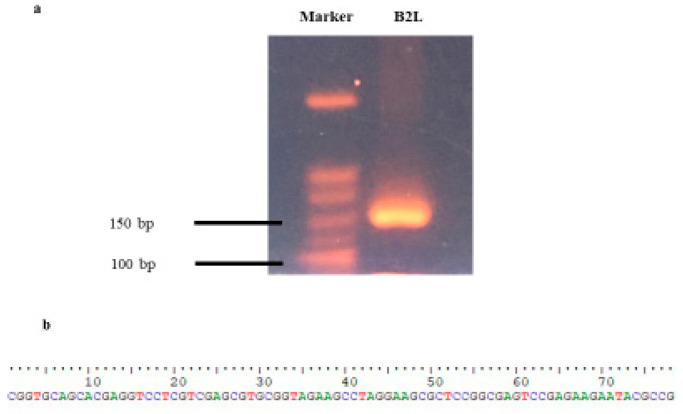
(**a**) The DNA product was run on 2% agarose gel. The size of the DNA products was 147 bp and (**b**) the DNA sequence specific for B2L gene was confirmed using next generation sequencing (marker is Bio-25 bp DNA ladder).

The genomic DNA was extracted from the skin tissue slides. The extracted genomic DNA was used for PCR amplification using primers forward 5′-CGGTGCAGCACGAGGTC-3′, and reverse 5′-CGGCGTATTCTTCTCGGACT-3′ according to Andreani et al. [4] with some modification. These primers are specific for the B2L gene, which encodes the major membrane protein of parapoxvirus. The primers were added to more base pairs (bp) (33 bp on the forward primer and 34 bp on the reverse primer) to add an adaptor sequence for the subsequent next generation sequencing. The adaptor sequence was following the instruction of Illumina guidance (Illumina, San Diego, CA, USA). Next generation sequencing (NGS) was performed on the iSeq 100 Sequencing Machine (Illumina, San Diego, CA, USA). The DNA product was run on 2% agarose gel and revealed that the size of the DNA products was 147 bp (80 + 33 + 34 bp) (Figure 2a). The DNA sequence specific for the B2L gene (80 bp) (Figure 2b) was confirmed using next generation sequencing. Thus, orf infection was diagnosed.

At a follow-up visit 2 weeks after initial presentation, all the noduloplaques on the scalp resolved with residual erythema and some crusts.

## 3. Discussion

We reviewed the literature and identified 11 manuscripts describing 12 individual cases with Orf lesions located on the head region (including face and scalp) [2,3,5,6,7,8,9,10,11,12,13], with the present case accounting for a 13th patient (Table 1). All these 13 cases except one [2] had either occupation or contact history. Eight cases had a single Orf lesion, but five cases (including our case) had multiple lesions [2,8,10,11]. Among those cases with multiple Orf lesions, two cases spread through the procedures (curettage, pulsed dye laser, and skin biopsy) used to treat or diagnose the primary Orf lesions [2,8]; one case was associated with the autoinoculation of Orf virus from the hand lesion [10]; and another case was linked to the human-to-human transmission of Orf virus from father to daughter via contaminated tweezers, which was used to squeeze acne lesions on the face [11]. Besides, trauma history might be a risk factor for primary Orf infection [3,6,11,13] or secondary spread [2,8]. In all 13 cases, 38.5% (5/13) had a history of trauma before the primary Orf lesion appeared and 53.8% (7/13) only had contact with infected animals or contaminated environments. As for our case, the patient denied any trauma or skin disease history on the scalp, nor previous Orf lesions on her hands. The histopathology of her scalp biopsy specimen also revealed no evidence of Hailey—Hailey disease. In addition, fortunately, no aggravation of the disease or increase in the lesion numbers was noted after skin biopsy.

Regional lymphadenopathy [3], generalized malaise [8], and mild fever [3,8] were possible accompanying systemic symptoms found in these cases. Among these 13 cases, 9 cases had skin lesions on the face region, but only 4 cases (including our case) had skin lesions on the scalp [2,3]. The sizes of the Orf lesions ranged from 0.7 cm to 5 cm in diameter, with most of them appearing to be at the targetoid or weeping stage of their natural course. Although the clinical appearance of the Orf lesions on the scalp were similar to the commonly reported Orf lesions on the hands, due to the unusual location of these lesions, they might be misdiagnosed as other skin diseases, such as pyogenic granuloma, malignant tumor, or anthrax [3,5,6,9], especially if no contact or occupation history was available. In our case, atypical infection was impressed at first before knowing the occupational contact history.

Although the diagnosis of Orf infection could be confirmed using contact history and clinical examinations in most of these cases, owing to the rare locations of the skin lesions on the head, the histological analysis [2,5,6,7,10,11], electron microscopy [3,5,6,8], or PCR [9,12] of the tissue specimen were still arranged to confirm the diagnosis. In our case, we used PCR analysis of the formalin-fixed paraffin-embedded skin biopsy specimens instead of fresh tissue because of initial uncertainty of the diagnosis when the skin lesion was biopsied.

Treatment was not administered in most of these cases because of the self-healing course of the Orf lesions. However, oral or topical antibiotics were prescribed in five cases (including our case) owing to the first impression of bacterial infection [2,8] or suspected secondary bacterial infection [10,13]. Laser treatment or surgical intervention, including excision, curettage, and cautery were performed in four cases [2,5,8,13], but three of them experienced exaggeration or persistence of the Orf lesions [2,8,13]. All these 13 cases finally had their Orf lesions resolved spontaneously in a few weeks. In our case, oral minocycline was prescribed because atypical bacterial infection could not be ruled out at the first visit and bacterial secondary infection should also be considered in the following treatment.

The diagnosis of Orf infection is usually confirmed clinically on the grounds of contact history with infected animals together with the characteristic skin lesions on exposed sites of the body. However, skin biopsy and other diagnosed tools, including virus culture, serological testing, transmission electron microscopy, and molecular testing such as PCR-based approaches and sequencing [1,4,14,15], can assist in the confirmation of the diagnosis in unusual cases. Nowadays, the diagnosis of acute Orf infection is based on PCR analysis of fresh samples (swabs, fluid from blister or pustule, or crusts). PCR analysis of paraffin-embedded tissue is useful when Orf was not considered at the time of skin biopsy [16]. However, not all laboratories routinely offer PCR analysis. Human employment status is an important risk factor for Orf infection, with veterinarians, butchers, farmworkers, and zookeepers considered to be highest risk groups [1]. After an incubation periods of from three to seven days, the Orf lesions typically appear through six clinical stages (maculopapular, targetoid, acute weeping, regenerative, papillomatous, and regressive stage), each lasting about one week and end in spontaneous resolution [17].

The lesions caused by the Orf virus can resemble other infections, such as the milker’s nodule, herpetic whitlow, anthrax, tularemia, sporotrichosis, or fish-tank granuloma. In our case, we examined the tissue Gram stain, fungal and mycobacterial culture to exclude these atypical infections. However, PCR technology to detect the DNA or RNA of these pathogens from fresh tissue specimens or paraffin-embedded tissue may be valuable supplements to culture and serology in doubtful cases. Other differential diagnoses include pyogenic granuloma and keratoacanthoma [1,14,18].

## 4. Conclusions

In our case, owing to the unusual location of the skin lesions on the scalp, the diagnosis of Orf infection might be difficult without access to accurate contact history. Further PCR analysis of the skin biopsy specimens also had an important role on confirming the diagnosis. Physicians should be aware that Orf infection occurs, albeit rarely, and should be considered it in the differential diagnosis of cases with relevant animal exposure.

## Figures and Tables

**Table 1:** Literature review of cases with Orf lesions located on the head region.

Author, Year	Age/Sex	Occupation	Contact History	Location	Clinical Features	Further Confirming Diagnostic Tools Except Skin Biopsy	Time to Resolution
Glass et al., 2009 [2]	10/M	Not mentioned	Not mentioned	Bilateral occipital scalp	A 0.9 cm bleeding nodule and another 2.5 cm ulcerated crusted nodule and the biopsied lesion recurred with an adjacent new lesion	No	Not mentioned
Rees et al., 1988 [3]	59/M	Mechanic of a farm	With trauma history	Right parietal scalp	A 2 × 3 cm pustular nodule. Lymphadenopathy in the posterior triangle of the neck	Electron microscopy	4 weeks
	61/M	Shepherd	With trauma history	Left parietal scalp	A 3 cm diameter haemorrhagic pustule Lymphadenopathy in the posterior triangle of the neck Fever (+)	Electron microscopy	4 weeks
Revenga et al., 2001 [6]	40/F	Shepherd	With trauma history	Right eyelid	A 2 cm crusted nodule	Electron microscopy	Weeks
Turk et al., 2014 [11]	16/F	Not mentioned	With trauma history	Frontotemporal region (face)	0.7–2.0 cm targetoid nodules	No	2 weeks
Gore Karaali et al., 2021 [13]	54/M	Not mentioned	With trauma history	Left eyebrow	A 3 cm edematous, ulcerated, hemorrhagic nodule	No	6 weeks
Mayet et al., 1997 [5]	61/M	Visitor of a farm	No trauma (contact with contaminated material in a shearing shed or the barbed wire of a fence)	Right temple	A 2 cm ulcerated nodule	Electron microscopy	Not mentioned
Gündüz et al., 2005 [7]	Not mentioned/M	Farmer	No trauma (fed animals)	Left mandible	Erythematous weeping nodule	No	8 weeks
Key et al. 2007 [8]	41/M	Farmer	No trauma (fed newborn lambs)	Right cheek	1. A 5 cm exophytic oozing crusted tumor, Cervical lymphadenopathy (+) Fever (+) 2. Lesions progressed after therapy (curettage and pulsed dye lase)	Electron microscopy	Weeks
Bayindir et al., 2011 [9]	19/M	Not mentioned	No trauma (contact with the animals or eating meat of sick animals)	Upper lip	Targetoid/weeping nodule	PCR	36 days
Duchateau et al., 2014 [10]	38/M	Not mentioned	No trauma (contact with a lamb)	Right hand and right lower jaw	A tender, erosive lesion and multiple papulonodules	No	8 weeks
Ata et al., 2017 [12]	52/M	Not mentioned	No trauma (contact with sheep with hands)	Nose	A painless 2 × 1.7 cm nodule	PCR	8 weeks
Our case	55/F	Farmer	No trauma (contact with a lamb)	Vertex and occipital scalp	Four 2 × 2 cm to 4 × 4 cm weeping and infiltrated noduloplaques	PCR	2 weeks

## Data Availability

The authors confirm that the data supporting the findings of this study are available within the article.
